# Case Report: Infantile Urticaria as a Herald of Neonatal Onset Multisystem Inflammatory Disease With a Novel Mutation in NLRP3

**DOI:** 10.3389/fimmu.2021.775140

**Published:** 2021-11-18

**Authors:** Anna E. Patrick, Eden M. Lyons, Lisa Ishii, Alan S. Boyd, Joseph M. Choi, Anna K. Dewan, Janet G. Markle

**Affiliations:** ^1^ Department of Pediatrics, Vanderbilt University Medical Center, Nashville, TN, United States; ^2^ Vanderbilt University School of Medicine, Nashville, TN, United States; ^3^ Department of Dermatology, Vanderbilt University Medical Center, Nashville, TN, United States; ^4^ Department of Pathology, Microbiology, and Immunology, Vanderbilt University Medical Center, Nashville, TN, United States

**Keywords:** Urticaria, NOMID, NLRP3, gene variant, pediatric autoinflammatory disease, inflammasome, IL-1 pathway, monogenic diseases

## Abstract

Neonatal multisystem onset inflammatory disorder (NOMID) is a severe autoinflammatory syndrome that can have an initial presentation as infantile urticaria. Thus, an immediate recognition of the clinical symptoms is essential for obtaining a genetic diagnosis and initiation of early therapies to prevent morbidity and mortality. Herein, we describe a neonate presenting with urticaria and systemic inflammation within hours after birth who developed arthropathy and neurologic findings. Pathologic evaluation of the skin revealed an infiltration of lymphocytes, eosinophils, and scattered neutrophils. Genetic analysis identified a novel heterozygous germline variant of unknown significance in the *NLRP3* gene, causing the missense mutation M408T. Variants of unknown significance are common in genetic sequencing studies and are diagnostically challenging. Functional studies of the M408T variant demonstrated enhanced formation and activity of the NLRP3 inflammasome, with increased cleavage of the inflammatory cytokine IL-1β. Upon initiation of IL-1 pathway blockade, the infant had a robust response and improvement in clinical and laboratory findings. Our experimental data support that this novel variant in *NLRP3* is causal for this infant’s diagnosis of NOMID. Rapid assessment of infantile urticaria with biopsy and genetic diagnosis led to early recognition and targeted anti-cytokine therapy. This observation expands the NOMID-causing variants in *NLRP3* and underscores the role of genetic sequencing in rapidly identifying and treating autoinflammatory disease in infants. In addition, these findings highlight the importance of establishing the functional impact of variants of unknown significance, and the impact this knowledge may have on therapeutic decision making.

## Introduction

Infantile urticaria is uncommon with underlying mechanisms including medication responses, infections, and rare mimickers that include autoinflammatory disease requiring immediate therapy. Neonatal multisystem onset inflammatory disorder (NOMID) is the severe form of a spectrum of disorders called cryopyrin-associated periodic syndromes (CAPS) that are caused by gain-of-function mutations in *NLRP3* (NLR family pyrin domain containing 3) (1). In NOMID, mutations in *NLRP3* result in increased inflammasome activity causing autoinflammation with inappropriately high secretion of IL-1β. Prompt disease recognition is critical for immediate therapeutic intervention to block the IL-1β pathway. We describe a neonate with urticaria and systemic inflammation that led to consideration of NOMID. A variant of unknown significance (VUS) was identified in *NLRP3*. We hypothesized that the VUS was pathologic and used histopathologic, genetic, and clinical features to evaluate this presentation.

## Case Description

A 4-day-old female, born at 35 weeks’ 6 days’ gestation, presented with a skin eruption hours after birth. Pregnancy course included Group B Streptococcus positivity and preterm labor. Family history was negative for autoimmune and autoinflammatory diseases.

**Figure 1:**
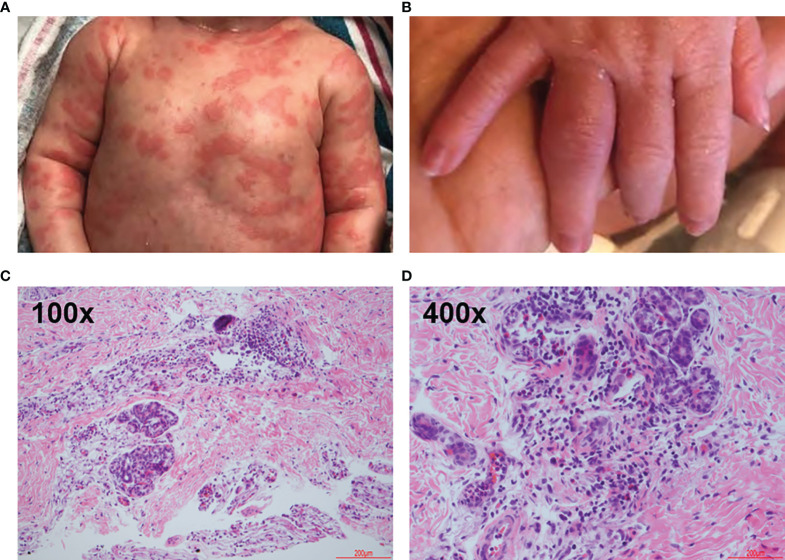
Clinical and Representative Histologic Findings. Infant at approximately 1 week of age. **(A)** Skin eruption on the ventral torso. **(B)** 4^th^ proximal interphalangeal joint arthritis. **(C, D)** Hematoxylin-eosin stains showing mixed dermal inflammation with numerous eosinophils. Skin, left leg biopsy. Step level sections with unremarkable dermis. Papillary dermal edema and a dense perivascular, periadnexal and interstitial infiltrate of lymphocytes and numerous eosinophils. No vasculitis.

Physical exam revealed a comfortable and afebrile neonate with well-defined, annular and polycyclic, blanchable erythematous macules coalescing into patches on the trunk, upper extremities, and lower extremities. On the face, neck, trunk, and upper extremities were confluent, erythematous, blanchable macules and polycyclic patches (**Figure 1A**). On the lower extremities were erythematous, blanchable macules and patches. The patches were migratory with no single lesion persisting more than 24 hours. The eruption waxed and waned with no identifiable trigger. Labs were significant for an elevated C-reactive protein (CRP) of 158 mg/L (reference: 0.3-6.1 mg/L) and erythrocyte sedimentation rate (ESR) of 50 mm/hr (reference: 2-37 mm/hr). Antinuclear antibody, anti-Ro/SSA, and anti-La/SSB were negative. Infection was considered and infectious studies were negative. Vision and hearing exams were normal.

After several days, arthritis developed in the right 4^th^ and left 2^nd^ proximal interphalangeal joints (**Figure 1B**). The eruption improved spontaneously, and CRP improved but did not return to normal. A genetic test for autoinflammatory disease was sent and the infant discharged home.

Genetic testing identified a heterozygous VUS in *NLRP3*. No challenges to genetic testing were encountered. During outpatient follow-up, the infant had recurrent urticaria and irritability and was admitted to the hospital. The infant had aseptic meningitis, transaminitis, and systemic inflammation. Anti-cytokine therapy with anakinra, an IL-1 receptor antagonist, was initiated. Within 24 hours the skin eruption and symptoms improved. The infant continued daily anakinra with good response.

## Timeline

**Figure 2:**
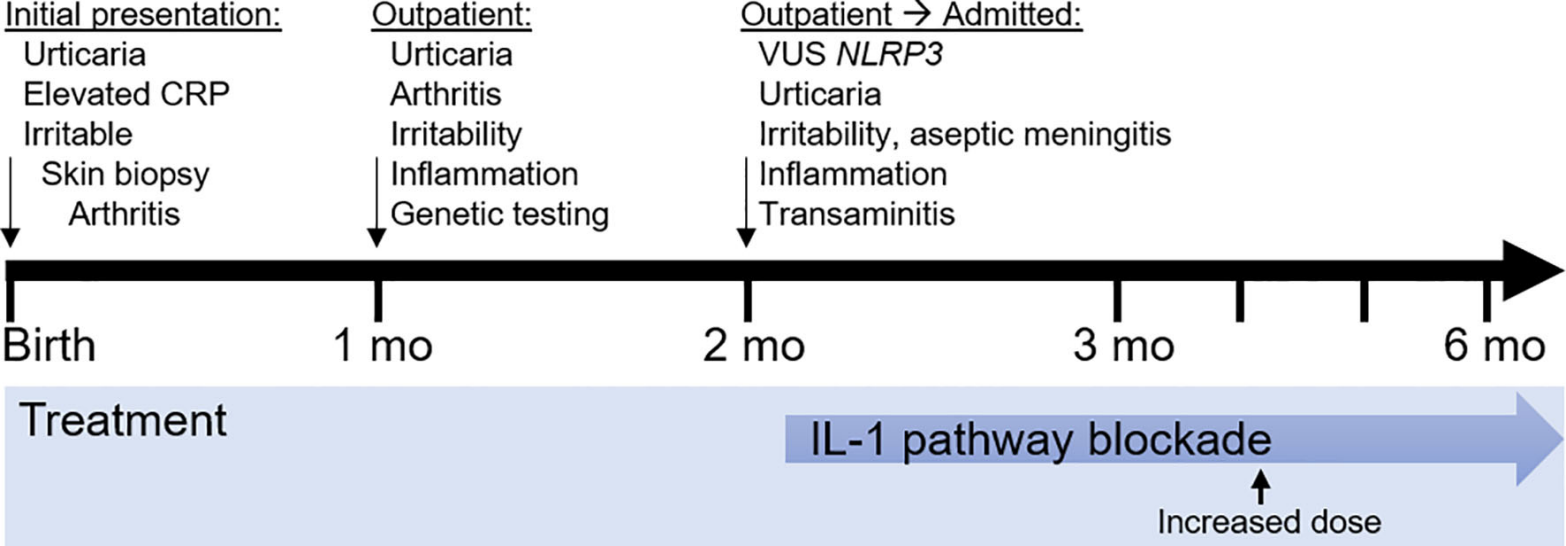
Timeline of clinical events and treatment strategies. mo, month.

The timeline is presented in **Figure 2**.

## Diagnostic Assessment

The diagnosis of NOMID was suspected given the patient’s presentation. During the initial admission, the pathologic evaluation included hematoxylin-eosin stain of a punch biopsy of the left leg demonstrated papillary dermal edema, dense perivascular, periadnexal, and interstitial infiltrate (**Figure 1C**). The perieccrine infiltrate included lymphocytes, numerous eosinophils, and scattered neutrophils (**Figure 1D**).

**Figure 3:**
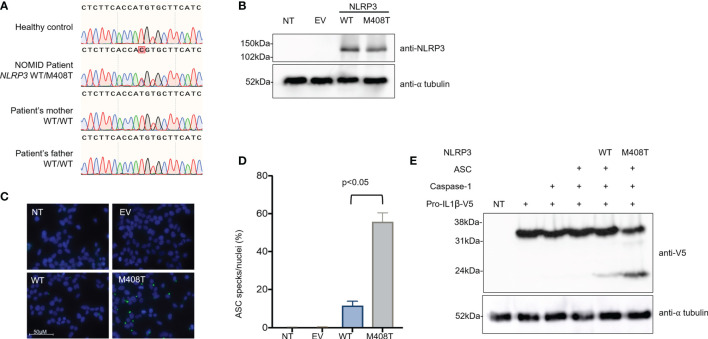
The heterozygous *de novo NLRP3* mutation M408T causes a gain of function for inflammasome activation. **(A)** Genomic DNA from a healthy control, the NOMID patient, and each of his parents was collected. PCR was used to amplify a region of NLRP3 surrounding the patient’s mutation, and these PCR amplicons were subjected to Sanger sequencing using a second set of specific primers. A T>C single nucleotide change, causing the missense mutation M408T in NLRP3 protein, was confirmed to be heterozygous in the NOMID patient and absent from each parent, indicating this is a *de novo* mutation. **(B)** Plasmids were constructed, encoding an empty vector (EV), wild-type NLRP3 (WT), or the NOMID patient’s NLRP3 mutation (M408T). HEK293T cells were either left non-transfected (NT) or were transfected with these plasmids, and whole cell lysates were used for western blotting. Using an antibody specific for NLRP3, expression of both WT and M408T NLRP3 protein was observed at the expected size (118kDa) with no notable difference in expression resulting from the mutation. Alpha-tubulin is used as a protein loading control. **(C)** HEK293^ASC-YFP^ cells were plated on coverslips, then were either left non-transfected (NT), or transfected with an empty vector (EV) or plasmids encoding WT or M408T NLRP3. Cells were stained with DAPI, mounted and visualized by fluorescence microscopy. Images are representative of 8 or more fields per condition. **(D)** Summary of ASC specks per nuclei for the experiment presented in **(C)**, and replicate experiments (n=4). P value is from a Mann-Whitney t-test. **(E)** HEK293T cells were either left non-transfected (NT, lane 1) or were transfected with the indicated combinations of plasmids encoding pro-IL-1β-V5, Caspase-1, ASC, and WT or M408T versions of NLRP3. After 8 hours, whole cell lysate was prepared and used for western blotting. Pro-IL1β is expected to migrate at 35.8kDa, while the mature cleaved form of IL-1β is 22.4kDa.

To assess for monogenetic autoinflammatory disease, a genetic evaluation was pursued. The Invitae Autoinflammatory Syndromes Panel identified a heterozygous VUS in *NLRP3* c.1223T>C (p.M408T). This variant was covered at a read depth of >1000 and variant reads represented ~50% of total reads, suggesting this is a germline variant. The M408T variant is not in the gnomAD v2 or v3 databases, comprising >200,000 individuals (2). M408 is a strictly conserved amino acid in the NLRP3 NACHT domain. This domain contains other NOMID-causing variants, including at positions 405 and 406 (3). Sanger sequencing of genomic DNA from the patients’ parents demonstrated the M408T mutation was *de novo* in this patient (**Figure 3A**).

To determine the impact of NLRP3 M408T on protein function, functional studies were pursued. The M408T mutation impact on NLRP3 expression and function was assessed by transfecting plasmids encoding the wild-type (WT) or M408T versions of NLRP3 into HEK293T cells. Whole cell lysates harvested 24hrs after transfection were analyzed by SDS-PAGE western blot with anti-NLRP3 antibody. No difference in the WT and M408T protein amounts were observed (**Figure 3B**). These plasmids were expressed in a HEK293^ASC-YFP^ cell line (4) and formation of ASC specks, a marker of inflammasome activation, were visualized by fluorescence microscopy (**Figure 3C**). Cells transfected with M408T showed significantly increased ASC specks per nuclei relative to WT (**Figure 3D**). Enhanced inflammasome activation was confirmed by co-expression of inflammasome components NLRP3, ASC, Caspase-1, and C-terminally V5-tagged Pro-IL1β in HEK293T cells. Eight hours post-transfection, western blot with anti-V5 antibody demonstrated increased cleavage of Pro-IL1β-V5 (35.8kDa) to the mature IL1β-V5 (22.4kDa) by inflammasomes containing M408T NLRP3, compared to WT NLRP3 (**Figure 3E**). These data establish M408T as a novel gain-of-function mutation in NLRP3.

**Figure 4:**
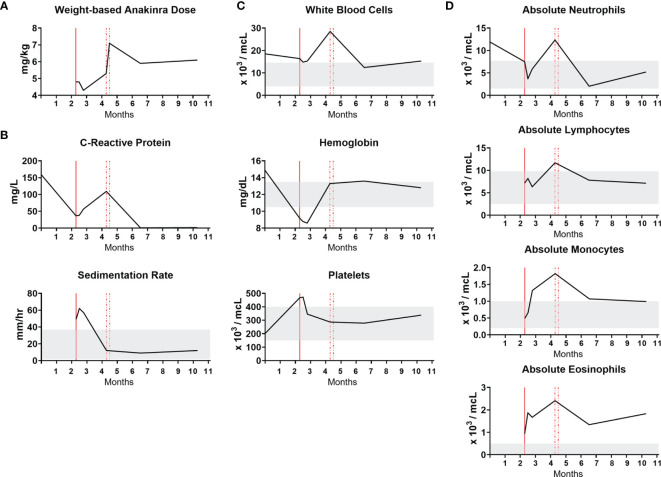
NOMID infant response to anakinra. The x-axis is a time course in months. Vertical red lines designate initiation and then dose increases of anakinra. Gray bands indicate normal values. **(A)** Weight-based anakinra dosing over time. **(B)** Markers of inflammation, CRP and ESR. CRP reference range is 0.3-6.1 mg/L. **(C)** Complete blood count values. **(D)** White blood cell differential absolute values.

To determine the clinical response to IL-1β pathway blockade, laboratory studies were monitored during treatment. The time course in months showing initiation of IL-1β pathway blockade with anakinra along with clinical laboratory results are shown (**Figure 4**). Anakinra, which competitively binds the IL-1 receptor to inhibit IL-1β signaling, is prescribed by weight-based dosing that is crucial for a growing infant (**Figure 4A**). Markers of inflammation, CRP and ESR, remained elevated after initiation of anakinra and improved upon dose increase (**Figure 4B**). Clinical CBC values, including white blood cells, hemoglobin, and platelets, returned to normal upon dose increase. The white blood cell differential highlights that while neutrophils, lymphocytes and monocyte levels normalize with increasing IL-1β blockade, eosinophils remain persistently elevated (**Figures 4C, D**). There were no adverse events related to IL-1β blockade.

## Discussion

Based on the clinical findings, pathology, genetic studies and experimental support, the infant is diagnosed with NOMID caused by the pathologic mutation M408T in *NLRP3*. A limitation of our study is that a single patient was identified with this mutation. NOMID is the most severe phenotype of CAPS, a spectrum of diseases that includes familial cold autoinflammatory syndrome (FCAS) and Muckle-Wells syndrome (MWS). The diagnostic criteria of CAPS include elevated inflammatory markers, including CRP or serum amyloid A during episodes, and two of the following: urticarial eruption; episodes triggered by cold or stress; sensorineural hearing loss; arthritis, arthralgia, and/or myalgia; chronic aseptic meningitis; and typical bone lesions (5). NOMID is distinguished from FCAS and MWS by long-term urticaria, neonatal onset, and neurologic involvement including intellectual disability and aseptic meningitis (6, 7). The clinical spectrum of NLRP3-related diseases is broad, and also includes a variant (R918Q) that causes an autosomal dominant syndrome including sensorineural hearing loss, with anakinra-responsive atypical CAPS present in some but not all patients (8). The role of NLRP3 hypermorphic alleles in autoinflammatory disease has also been confirmed in a mouse model that recapitulated a human MWS-causing mutation (9). Our case highlights many classic features of NOMID and the importance of considering early genetic diagnosis with heightened concern for VUS in critical genes like *NLRP3*.

NOMID is caused by gain-of-function mutations in *NLRP3*, which encodes for cryopyrin, an inflammasome protein (6). Germline and somatic variants can cause NOMID, highlighting genetic heterogeneity in this disease (1, 10). Urticaria can be one of the earliest signs of NOMID, with over 90% patients displaying this feature (11). Histopathology relatively unique to the urticarial lesions of CAPS include interstitial infiltrate containing neutrophils extending into adnexal structures, which is highly sensitive and specific to neutrophilic urticarial dermatoses (12). This neutrophilic epitheliotropism, especially the perieccrine infiltrate, distinguishes CAPS from conventional urticaria. Our case displayed this relatively unique histopathology feature, which first raised the concern for NOMID.

NOMID treatment includes IL-1 pathway blockade. The IL-1 receptor antagonist anakinra is the FDA approved drug for NOMID treatment. Monocytes from patients with CAPS due to hypermorphic mutations of NLRP3 were reported to secrete higher levels of IL-1β in response to LPS stimulation, compared to monocytes from healthy controls (13, 14). However, this cellular phenotype was abrogated in patients being treated with IL-1 blocking therapy (13). Before IL-1 blocking therapies, over 20% of NOMID patients died before adulthood (15). Early treatment with anakinra rapidly improves inflammation and multiorgan involvement (11, 16). Patients are generally followed by a pediatric rheumatologist with routine labs to monitor inflammation and end organ dysfunction, with improvements in inflammatory marker indicating an effective response to anakinra therapy (11).

In summary, we present the case of a neonate with urticaria complicated by systemic inflammation, arthritis, aseptic meningitis who had clinical improvement in response to targeted anti-cytokine therapy with an IL-1 receptor antagonist. The *NLRP3* M408T mutation was determined to be a pathologic variant. Early recognition of neonatal urticaria warrants consideration for NOMID. Rapid workup including biopsy, lab studies and genetic evaluation are essential to initiation of targeted therapies and prevent multiorgan damage.

## Patient Perspective

The patient adhered to the treatment proposed. Family was satisfied by the improvement in the clinical condition and ability to maintain medical care at home.

## Data Availability Statement

The original contributions presented in the study are included in the article/supplementary material. Further inquiries can be directed to the corresponding author.

## Ethics Statement

The studies involving human participants were reviewed and approved by Institutional Review Board, Vanderbilt University Medical Center. Written informed consent to participate in this study was provided by the participants’ legal guardian/next of kin. Written informed consent was obtained from the minor(s)’ legal guardian/next of kin for the publication of any potentially identifiable images or data included in this article.

## Author Contributions

AP, AD, and JM contributed to conception and design of the study. AB performed dermatopathology studies. AP, JC, and JM performed experiments and statistical analysis. AP, LI, and AD were clinicians involved in patient care. AP, EL, LI and JM wrote the manuscript and prepared figures. All authors reviewed the manuscript. All authors contributed to the article and approved the submitted version.

## Funding

This work was supported by the National Institutes of Health with K12HD087023 (AP) and a pilot and feasibility award from P30DK058404 (JM) and the Vanderbilt Human Immunology Discovery Initiative (AP, JM).

## Conflict of Interest

The authors declare that the research was conducted in the absence of any commercial or financial relationships that could be construed as a potential conflict of interest.

## Publisher’s Note

All claims expressed in this article are solely those of the authors and do not necessarily represent those of their affiliated organizations, or those of the publisher, the editors and the reviewers. Any product that may be evaluated in this article, or claim that may be made by its manufacturer, is not guaranteed or endorsed by the publisher.
